# B7-H7 knockdown suppresses the proliferation, metastasis, and drug resistance of B-cell non-Hodgkin lymphoma cells by inhibiting the PI3K/Akt pathway

**DOI:** 10.3389/fonc.2025.1665309

**Published:** 2025-10-16

**Authors:** Aili Zhai, Qing Zou, Jiawei Yin, Shixin Ge, Xingfang Xiong, Tao Yan, Xiaoli Xie, Lijuan Wang

**Affiliations:** ^1^ Graduate Training Base of Jinzhou Medical University (Linyi People’s Hospital), Linyi, Shandong, China; ^2^ Department of Hematology, Linyi People’s Hospital, Linyi, Shandong, China; ^3^ Laboratory Animal Center, Linyi People’s Hospital, Linyi, Shandong, China; ^4^ The 11th Clinical College of Qingdao University (Linyi People’s Hospital), Linyi, Shandong, China; ^5^ Central Laboratory, Linyi People’s Hospital, Linyi, Shandong, China; ^6^ Linyi Key Laboratory of Tumor Biology, Linyi, Shandong, China; ^7^ Key Laboratory for Translational Oncology, Xuzhou Medical University, Xuzhou, Jiangsu, China

**Keywords:** B7-H7, B-NHL, rituximab, resistance, cancer progression, PI3K/AKT

## Abstract

**Background:**

The activation of key immune regulatory factors is an important driving factor in the progression of therapeutic resistance in patients with B-NHL. As a novel immune regulatory factor of the B7 family, surface B7-H7 interacts with receptors on active T cells to promote tumor immune escape. However, investigations of B7-H7 on B-NHL are still lacking.

**Methods:**

*In vitro* assays were performed to examine the proliferation, migration, invasion, and starvation tolerance abilities of B7-H7 knockdown cells. A xenograft mouse model of B-NHL was established to investigate the *in vivo* functions of B7-H7. The impact of B7-H7 on patient prognosis was analyzed using the GEPIA and GEO datasets. We also examined whether B7-H7 knockdown affects resistance to rituximab (RTX) and verified this by establishing a rituximab-resistant cell line (RRC). Finally, RNA sequencing was performed on B7-H7 knockdown cells, rituximab-treated cells, and rituximab-resistant cells.

**Results:**

B7-H7 knockdown inhibited tumor growth *in vivo* and suppressed cell proliferation, migration, invasion, and starvation-bearing ability *in vitro*. Patients with diffuse large B-cell lymphoma (DLBCL) with high expression of B7-H7 showed an increased death rate. B7-H7 knockdown increased sensitivity to rituximab, and the strong resistance of RRC was weakened to some extent when B7-H7 expression in RRC was inhibited. RNA sequencing revealed that the PI3K/Akt pathway may play an important role in B7-H7 knockdown in cells and drug-resistant strains. This was confirmed by western blotting and agonist/inhibitor treatment.

**Conclusions:**

Suppression of B7-H7 inhibited tumor progression and induced RTX sensitivity by suppressing the PI3K/Akt pathway.

## Introduction

B-cell non-Hodgkin lymphoma (B-NHL) is a common malignant tumor with a heterogeneous origin in the blood system, accounting for approximately 85% of all lymphomas. B-NHL progresses rapidly with a high degree of malignancy (1). The primary treatments for B-NHL include chemotherapy, radiotherapy, immunotherapy, and hematopoietic stem cell transplantation. Combined therapy with rituximab (RTX) and cyclophosphamide, doxorubicin, vincristine, and prednisone (CHOP), the standard preferred therapy for B-NHL, can significantly improve the chances of cure and overall survival of patients. However, approximately 50% of patients do not initially respond to this treatment regimen, and the majority eventually develop resistance to further treatment with RTX (2). The expression and activation of key immune checkpoints are among the most important considerations for the emergence of therapeutic tolerance in patients with B-NHL.

Tumor immunotherapy is primarily based on the induction and activation of tumor-specific cytotoxic T cells to clear metastatic tumor cells. The synergistic stimulation signal is produced by a combination of B7 family molecules expressed on the surface of antigen-presenting cells and their respective receptors expressed on T cells, such as B7–1 with CD28 and B7–2 with cytotoxic T lymphocyte-associated antigen-4 (CTLA-4); this is known as the classical B7 pathway (3). In our previously published article (4), we reviewed the research progress on all B7 family molecules in lymphoma.

B7-H7, also called Human endogenous retro virus-H long terminal repeat-associating 2 (HHLA2), is a novel type I transmembrane glycoprotein of the B7 family (5). Recent studies have shown that multiple tumors express B7-H7 and that the degree of B7-H7 expression is closely related to the clinical and biological characteristics of tumors, such as tumor stage, tumor size, degree of tumor infiltration, lymph node metastasis, distant metastasis, and overall survival of patients (6–11). The widespread expression of B7-H7 in tumors and its clinical significance suggest that B7-H7 may have a major influence on tumor development by inhibiting immune responses. Research has shown that B7-H7 expressed on the surface of tumor cells may promote tumor immune escape by interacting with receptors on activated T cells, thereby promoting the occurrence and development of tumors (12). However, the mechanisms underlying the interaction between B7-H7 and immune cells, as well as its relationship with tumor drug resistance, remain unclear. Thus, the appropriate immunotherapeutic approach for targeting B7-H7 has not been established. In addition, to date, research on B7-H7 has focused on solid tumors, with almost no studies on hematological tumors.

RTX is a monoclonal antibody targeting CD20 that induces cytotoxicity and mobilizes the host phagocytic immune cells through the Fc-γ receptor (13). RTX is presently included in all stages of B-NHL treatment, including first-line, maintenance, and rescue treatments. However, most patients ultimately develop resistance to further RTX treatment, hindering its clinical application. Therefore, to improve the current clinical treatment status, recognizing the origin of recurrence and drug resistance after B-NHL treatment and identifying the key immune regulatory factors leading to the development of drug resistance hold much value. The phosphatidylinositol 3-kinase/protein kinase B (PI3K/AKT) signaling pathway is highly active in many cell types, and numerous small molecule inhibitors targeting the PI3K/AKT pathway have been developed for cancer treatment. However, clinical applications have revealed that using PI3K or AKT inhibitors alone does not achieve sustained inhibition. Therefore, combination therapy involving PI3K or AKT inhibitors alongside chemotherapeutic agents is often employed to enhance clinical treatment efficacy (14).

In summary, the extensive research on B7-H7 in solid tumors has yielded insights into B-NHL because of its complex types and high rate of incidence, intractability, and recurrence. In this study, we evaluated the impact of B7-H7 on the proliferation, metastasis, and drug tolerance of B-NHL cells through *in vitro* experiments, mouse models, and RNA sequencing (RNA-seq) analysis. The findings will provide a theoretical basis and new targets for clinical treatment, contributing to the development of precision medicine for B-NHL.

## Materials and methods

### Cell culture and lentivirus infection

The Raji cell line (RRID: CVCL_0511), which belongs to the B-NHL category, was obtained from the Cell Bank of the Chinese Academy of Sciences (Shanghai, China), and STR identification was performed. All cell cultures underwent routine mycoplasma testing to confirm the absence of contamination. The cells were maintained in complete RPMI-1640 medium (C11875500BT, Gibco, USA) containing 10% fetal bovine serum (FBS) (10270106, Gibco, USA) and 1% penicillin-streptomycin double antibiotic mixture (SV30010, Hyclone, USA) in a CO_2_ cell incubator. Human B7-H7-targeting and control short-hairpin RNA (shRNA) lentiviral particles were purchased from Santa Cruz (sc-78498-V, sc-108080; California, USA). Raji cells were diluted to a density of 1 × 10^6^ cells/well and transfected with shRNA lentiviral particles. After 48 h, transfected cells were then selected using 1 μg/mL puromycin (P8230-25; Solarbio, Beijing, China). The cells were divided into two groups according to the transfected lentiviral particles: cells transfected with control shRNA lentiviral particles (Lv-NC group) and those transfected with B7-H7-shRNA lentiviral particles (Lv-B7-H7 group). To ensure stable selection, transduced cells were subsequently maintained in puromycin-containing medium throughout all subsequent cultures. Two groups of Raji cells were collected for RNA extraction, protein extraction, and flow cytometry to verify knockdown efficiency.

### B7-H7 mRNA expression detection

Cells were obtained, and TRIzol reagent (15596018, Invitrogen, USA) was used for RNA extraction. cDNA was synthesized under the following conditions: 42°C for 2 min, 37°C for 15 min, and 85°C for 5 s with Hiscript Il RT SuperMix (R323-01, Vazyme, Nanjing, China). The conditions for qRT-PCR were as follows: pre-denaturation at 95°C for 10 min, 95°C for 15 s, 60°C for 1 min, repeated for 40 cycles with Taq Pro Universal SYBR qPCR Master Mix (Q712-02, Vazyme, Nanjing, China) on an ABI 7500 PCR system (Waltham, USA). The primer sequences used in our study were as follows: *B7-H7*-F, 5′-ACC CGT GAT GAA GTA TGA AA-3′; *B7-H7*-R, 5′-AAA GAA TCC AAA GAC CCT GT-3′. The GAPDH primers were consistent with the primer sequences used in our previous study (15). These primers were generated by Sangon Biotech (Shanghai, China). The mRNA expression levels of B7-H7 were converted relative to *GAPDH* levels after applying a comparative formula (2^-△△CT^).

### Western blot analyses

Cells were collected and lysed with radioimmunoprecipitation assay (RIPA) lysis solution (YK2233; Y&K Bio, Xi’an, China). Quantitative assays were performed using a BCA kit (YK2237, Y&K Bio, Xi’an, China). The detection amount for each sample was 15 μg. The protein electrophoresis conditions were as follows: 5% concentrated gel electrophoresis for 25 min at 80 V and 10% separating gel electrophoresis for 70 min at 120 V. The proteins were transferred to a 0.22-μM PVDF (ISEQ00010, Millipore, USA) membrane at constant current (400 mA) for 120 min. The membranes were blocked with skim milk (232100; BD-Difco, BD, USA) for 1-1.5 h and incubated with primary antibodies overnight at 4 °C and then 1.5 h at room temperature. The membranes were then incubated with the secondary antibodies for 1 h at room temperature after washing three times with Tris-buffered saline with Tween-20. Finally, the membranes were imaged using a chemiluminescence imaging instrument (Tanon 5200multi, Shanghai, China). The analyses were performed using primary polyclonal antibodies against B7-H7 (RRID: AB_2876363, 1:500 dilution, ab214327, Abcam, USA), PI3K (RRID: AB_11042594, 1:1000 dilution, 60225-1, Proteintech, USA), phospho-PI3K (RRID: AB_659940, 1:1000 dilution, 4228s, Cell Signaling Technology, USA), and GAPDH (RRID: AB_2107436, 1:5000 dilution, 60004-1, Proteintech, USA) and secondary goat-anti-mouse (1:10000 dilution, YK2232, Y&K Bio, Xi’an, China) and goat-anti-rabbit (1:10000 dilution, YK2231, Y&K Bio, Xi’an, China) antibodies. The relative expression level of the target protein was calculated by normalizing its density to GAPDH, followed by standardization using the calibrated values from the control group.

### Flow cytometry

Both groups of Raji cells were washed with phosphate-buffered saline (PBS) after collection. The cells were gently stirred back into a suspension containing 100 μL of blocking solution (PBS containing 5% FBS) and stained with allophycocyanin-labeled monoclonal antibody against B7-H7 (clone: MA57YW, RRID: AB_2784652, 5 μL/test, 17-6537-42, Ebioscience, USA) at ambient temperature in the dark for 30 min to detect the surface protein expression of B7-H7. Cells were detected using a Canto II flow cytometer (BD Biosciences, USA) and further analyzed using FlowJo software (RRID: SCR_008520).

### Subcutaneous xenograft model and immunohistochemical analyses

Six-week-old female non-obese diabetic/severe combined immunodeficiency (NOD/SCID) mice were purchased from Beijing Vital River Laboratory Animal Technology Co., Ltd. (Beijing, China). *In vivo* studies were approved by The Institutional Animal Care and Use Committee of Lunan Pharmaceutical Group Co., Ltd. (approval number: AN-IACUC-2021-061). All methods were carried out in accordance with relevant guidelines and regulations. Mice were randomly divided into two groups (n = 8 per group). All mice were bred and maintained in a SPF barrier facility. And the mice were maintained on a 12/12-hour light/dark cycle, 20-26°Cwith abundant sterile pellet food and waste. Two groups of Raji cells were mixed with Matrigel (40183ES08; YEASEN, Shanghai, China) at a volume ratio of 1:1, and 0.1 mL of the mixture was inoculated subcutaneously into the right flank of each NOD/SCID mouse. The number of Raji cells inoculated into each mouse was 5 × 10^6^. Tumor volume was measured every three days by an experimenter blinded to the injection conditions and experimental cohort. On the 39th day, the mice were anesthetized with sodium pentobarbital and their tumor tissues were excised, weighed, and captured using a camera. The tumor tissues were fixed in 4% polyformaldehyde for immunohistochemistry (IHC). IHC was performed using a two-step universal immunohistochemical detection kit (PV-9000; ZSGB-BIO, Beijing, China) according to the manufacturer’s instructions. The tissues were cut into small pieces, dehydrated, and embedded in a wax pool overnight. Paraffin slices were cut and dried before dewaxing and hydration. Next, antigen repair and blocking were performed, followed by incubation with 100 μL of the primary antibody Ki-67 (RRID: AB_302459, ab16667, 1:200 dilution; Abcam, USA) for 1 h at 37°C. After incubation with the reaction enhancement solution for 20 min, samples were conjugated with the goat anti-mouse/rabbit IgG (PV-9000; ZSGB-BIO, Beijing, China) for 15 min at 37°C. Next, the slices were stained with 3,3′-diaminobenzidine (DAB) solution (ZLI-9017; ZSGB-BIO, Beijing, China) at room temperature in the dark for 5 min, followed by counterstaining with hematoxylin and dehydrate. After drying, tree glue was added, and images were photographed using a microscope (Olympus, Japan) at least three microscopic fields. Mean integrated optical density (IOD) per unit area values were automatically calculated using Image-Pro Plus (RRID: SCR_007369).

### Cell counting kit-8 assay

Cells were seeded in 96-well plates (20,000 cells/well). Next, 10 µL of cell counting kit 8 (CCK-8) solution (BB-4202; Bestbio, Shanghai, China) was added at different times according to the experimental setup and maintained for 3 h. The absorbance of each well was measured at 450 nm by using a microplate reader (SpectraMax M5, USA). All cell viability data presented in this study represent relative values calculated through a two-step normalization process: First, background absorbance (measured from media-only wells) was subtracted from all wells. Subsequently, the adjusted absorbance values were normalized against the calibrated control group.

### EdU-488 incorporation and immunofluorescence assays

The EdU-488 incorporation assay was performed using an EdU-488 cell detection kit (C0071S, Beyotime, Beijing, China) according to the manufacturer’s instructions. Cells belonging to the two groups were plated in a 6-well dish (2 × 10^5^ cells per well) and incubated with 2 μM EdU for 1 h in a cell incubator. The cells were harvested and resuspended with 30 μL of PBS. Cell suspensions were dropped onto adhesive slides and allowed to dry naturally to form a single-cell layer. The cells were fixed in 4% polyformaldehyde (P1110; Solarbio, Beijing, China), permeabilized with 0.4% Triton-X (IT9100; Solarbio, Beijing, China), and blocked with 5% bovine serum albumin (BSA) (SW3015; Solarbio, Beijing, China). Next, a 488 click reaction solution was prepared and added onto the slides, followed by labeling at ambient temperature in the dark for 30 min. The cells were stained with Hoechst 33342 (1×) for 10 min in the dark. Images were obtained using a fluorescence microscope (ECLIPSE Ti Microscope; Nikon, Japan). Hoechst 33342 was stimulated with ultraviolet light and the 488 dye was stimulated with blue light. All images were counted and calculated using Image-Pro Plus software. The operation steps for the immunofluorescence analysis were similar to those of the EdU-488 incorporation assay, except for EdU incubation and the 488 click reaction. Instead, primary antibody Ki-67 (1:1000 dilution, ab16667; Abcam, USA) and Cy3-labeled goat anti-rabbit IgG (A0516; Beyotime, Beijing, China) staining were performed after blocking for 2 h at 37°C. The Cy3 dye was stimulated with a green light.

### Cell migration and invasion assays

The 24-well 8.0-μm pore size Transwell system (Falcon, Corning, NY, USA) was used as the top chamber for the migration assay, and the 24-well Matrigel Invasion Chamber (Biocoat, Corning, NY, USA) containing pre-coated Matrigel was used for the invasion assay. The remaining steps of the invasion assay were the same as those used in the migration assay. The top chamber was supplemented with 200 μL of serum-free medium containing 1% BSA and including a total of 2 × 10^5^ cells. The lower chamber (Falcon, Corning, USA) was supplemented with 700 μL of complete medium. After 24 h, the top chambers were removed, and a cotton swab was used to remove non-migrating and non-invading cells. The top chambers were immersed in 4% paraformaldehyde (P1110; Solarbio, Beijing, China) and fixed for 20 min, and stained with 0.1% crystal violet (G1062; Solarbio, Beijing, China). The top chambers were imaged under an ECLIPSE Ti Microscope across five randomly captured fields. Migrated/invaded cells were manually quantified using Image-Pro Plus software.

### Cell-starvation assay and cell apoptosis measurements

Two groups of cells were seeded in a 6-well plate at a density of 3 × 10^5^ cells/well and maintained in serum-free 1640 medium for 48 h. An Annexin V-FITC/propidium iodide (PI) apoptosis detection kit (BB-4101; Bestbio, Shanghai, China) was used for cell apoptosis detection according to the manufacturer’s instructions. After 48 h, the cells were harvested and rinsed with PBS, and then gently stirred back into 400 μL of Annexin V binding solution. Next, 5 μL of Annexin V-FITC was added to the cell suspension, which was stored at 4°C for 15 min in the dark, followed by the addition of 10 μL of PI. Cells were immediately detected on a Canto II flow cytometer (BD, USA) and analyzed with FlowJo software.

### Caspase 3 enzyme activity detection

A caspase 3 activity detection kit (C1116; Beyotime, Beijing, China) was used to validate apoptosis. First, the absorbance of pNA (0, 10, 20, 50, 100, 200 μM) was tested at 405 nm, and a pNA standard curve was created. After starvation for 48 h, cells were collected and rinsed with PBS. The cells were maintained in lysis solution on ice for 15 min and centrifuged to collect the supernatant. Protein concentrations were determined using a Bradford protein concentration determination kit (P0006; Beyotime, Beijing, China) according to the manufacturer’s instructions. Next, a detection system was established in a 96-well plate that included sample and blank control wells. The plates were cultured in a cell incubator until the color changed. Finally, the absorbance of each well was measured at 405 nm.

### Gene expression profiling interactive analysis and gene expression omnibus database analysis

Survival analysis was performed using Gene Expression Profiling Interactive Analysis (GEPIA) (RRID: SCR_018294; http://gepia.cancer-pku.cn/index.html). We designated diffuse large B-cell lymphoma (DLBCL) patient samples and categorized them into low- and high-expression groups based on the B7-H7 expression levels (n = 23 for each group). Subsequently, disease-free survival analysis was conducted. GSE4475 (118 DLBCL samples) datasets were obtained from the Gene Expression Omnibus (GEO) database (RRID: SCR_005012, https://www.ncbi.nlm.nih.gov/geo/) for further analyses. Clinical information and expression levels were obtained using the R package (version 4.2.2). The samples were divided into two groups based on the quartile method, using the median expression level. Those with expression levels in the bottom 25% were classified as the low-expression group, while those in the top 25% were classified as the high-expression group.

### Rituximab sensitivity detection and construction of resistant cells

For the RTX (S28566; Yuanye, Shanghai, China) treatment, the cells were co-cultured with different treatment concentrations for different durations according to the experimental settings. However, all experiments involving RTX require 10% healthy human serum (NS). RTX sensitivity was measured using the CCK-8 assay as described above. The RTX-resistant cell line (RRC) was established using an increasing concentration gradient method. Initially, Raji cells were cultured in l μg/mL RTX containing 10% NS for 48 h, and then switched to fresh medium without RTX. When the cells reached the logarithmic growth phase, they were cultured with the next RTX concentration. The RTX concentrations used in the gradient were l μg/mL, 5 μg/mL, 10 μg/mL, 20 μg/mL, 50 μg/mL, and 100 μg/mL, respectively, and each concentration was repeated twice. Further experiments with the RRC were performed after withdrawal of RTX for at least one week.

### shRNA transfection

Knockdown of B7-H7 in the RRC was performed using transient shRNA transfection. shRNA targeting B7-H7 (shB7-H7) and a control shRNA (shNC) were designed and synthesized by GeneCopeia. The shRNA sequences used are listed in **Table 1**. The RRC was spread in 6-well plates at a density of 5 × 10^5^ cells/well and transfected with 3 μg of shRNA using FuGENE 6 transfection reagent (E2691, Promega, USA). After incubation for 48 h, the cells were collected, and the knockdown efficiency was determined as described above. For the RTX treatment experiment, the RRC was transfected with shRNA for 48 h before the addition of RTX.

**Table 1:** shRNA sequence.

shRNA	Vector	Sequence (5’-3’)
shRNA-B7-H7	LVRU6GP	GATCCGGCGTGTTAAGTGTTTATCCTCTCAAGAGCTCTTGAGAGGATAAACACTTAACACGCTTTTTGGAATT
shRNA-NC	LVRU6GP	GATCCGGCTTCGCGCCGTAGTCTTATCAAGAGCTCTTGATAAGACTACGGCGCGAAGCTTTTTGGAATT

### RNA-seq analysis

Six groups of Raji cells (Lv-B7-H7 vs. Lv-NC, Lv-B7-H7+RTX vs. Lv-NC+RTX, RRC vs. wt; three replicates per group) at a concentration of 2 × 106 cells were harvested and preserved in 1 mL of TRIzol reagent. All library synthesis and sequencing procedures were performed by Sinotech Genomics Co., Ltd. (Shanghai, China). After passing quality control checks, RNA samples were processed for library construction. Eukaryotic mRNA was enriched using Oligo(dT)-coated magnetic beads, followed by random fragmentation of mRNA with fragmentation buffer. Using the fragmented mRNA as template, the first strand of cDNA was synthesized with random hexamers. Subsequently, the second cDNA strand was generated through addition of buffer solution, dNTPs, and DNA Polymerase I. The double-stranded cDNA was then purified using AMPure XP beads. The purified cDNA underwent subsequent processing including end repair, adenylation (A-tailing), and adapter ligation. Fragment size selection was performed using AMPure XP beads, followed by PCR amplification to enrich the final cDNA library. Paired-end libraries were constructed with the TruSeq™ RNA Sample Preparation Kit (Illumina, USA) and then sequenced on Illumina NovaSeq 6000 (Illumina, USA). The raw image data files generated from high-throughput sequencing were processed through CASAVA base calling analysis to produce raw sequenced reads. These reads subsequently underwent stringent quality control checks and filtering procedures to obtain high-quality clean data. GRCh38.108 using Hisat2 (version 2.0.5) was used for mapping. Gene expression levels were expressed as fragments per kilobase of exons per million reads mapped (FPKM). All subsequent bioinformatics analyses were performed using R software. Differentially expressed genes (DEGs) were chosen according to the criterion value for the significance of differences (|fold change| value > 1.5, q value < 0.05) using the R package edgeR for further analysis. Kyoto Encyclopedia of Genes and Genome (KEGG) pathway enrichment (RRID: SCR_012773) was performed using the enriched R package. Gene Ontology (GO) analysis of biological processes, cellular components, and molecular functions was performed using the enriched R package.

### Blockage and activation of the PI3K/Akt pathway

Raji Lv-NC and Lv-B7-H7 cells were seeded in 96-well plates at a density of 20,000 cells/well. 740-YP (30 μM, S7865, selleck, USA) was added into the corresponding wells. The RRC was treated with 30 μM LY294002 (S1105, selleck, USA) for 30 min prior to incubation with RTX. The remaining steps were identical to those used in the CCK-8 assay.

### Statistical analysis

All data were processed and graphed by GraphPad Prism 5.0 (RRID: SCR_002798) and presented as mean ± standard deviation (mean ± SD). Survival analyses were conducted using log-rank tests and Cox regression models, while all other comparative analyses employed Student’s t-tests. A P-value of less than 0.05 (two-tailed) was considered to indicate statistically significant differences. All experiments were conducted at least three times.

## Results

### B7-H7 knockdown suppressed B-NHL tumor progression in the xenograft mouse model

**Figure 1:**
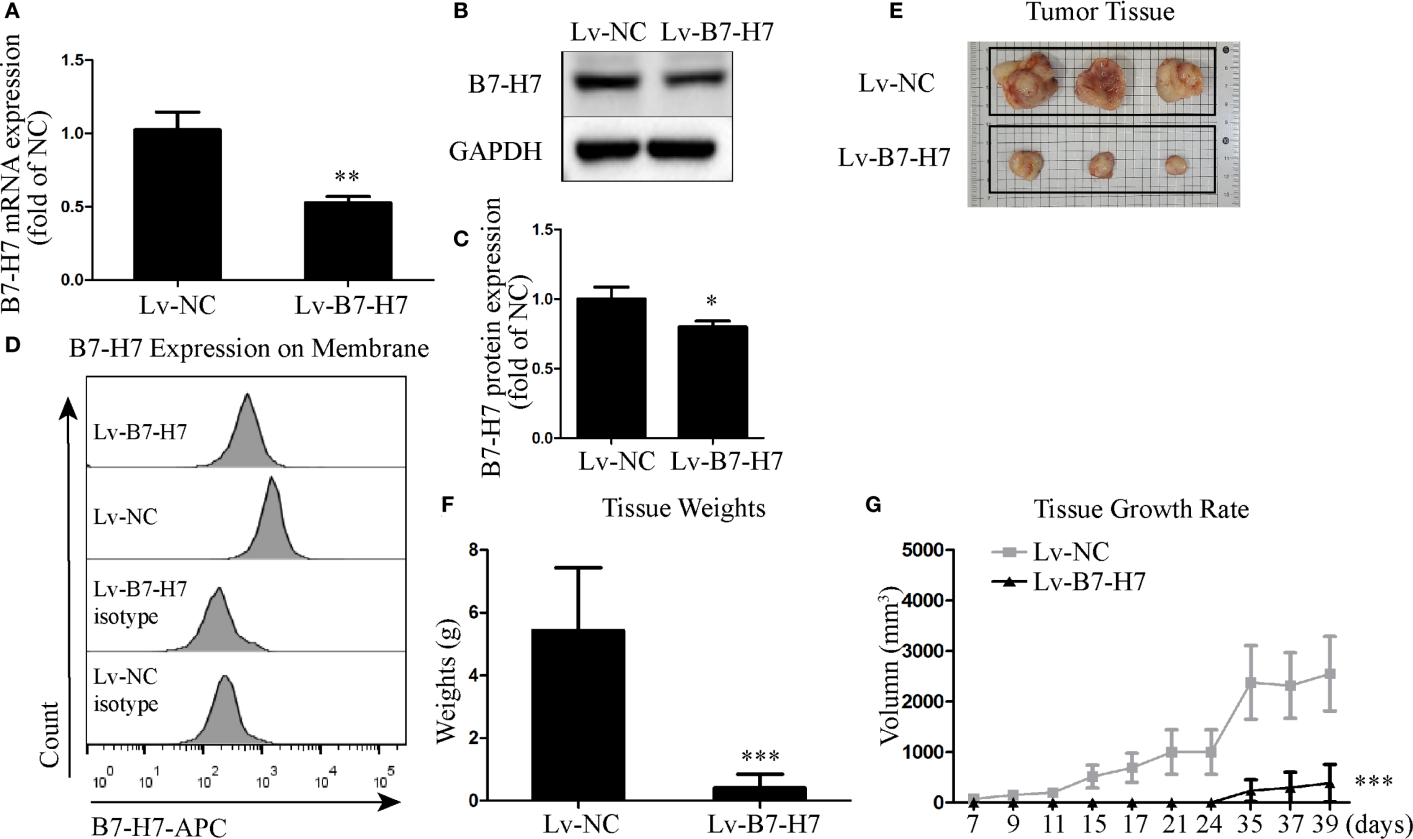
Knockdown of B7-H7 inhibited tumor progression in mice. **(A)** B7-H7 mRNA expression was detected by qRT-PCR. **(B, C)** B7-H7 protein expression was detected by western blotting. **(D)** Cell surface B7-H7 expression was determined by flow cytometry. **(E, F)** NOD-SCID mice were dissected after subcutaneous inoculation with the two groups of cells for 39 days, and the tumor tissue was completely dissected and weighed. **(G)** The tumor volumes were recorded every three days and a growth curve was drawn. *p < 0.05, **p < 0.01, ***p < 0.001.

To assess the role of B7-H7 in B-NHL, we knocked down the expression of B7-H7 by transfection with lentiviral shRNAs. We also examined the expression levels of B7-H7 using qRT-PCR, flow cytometry, and western blotting to verify the efficiency of the knockdown. As shown in **Figures 1A-D**, B7-H7 expression was significantly inhibited in Lv-B7-H7 cells in comparison with Lv-NC cells. The *in vivo* role of B7-H7 was investigated by constructing a subcutaneous xenograft model in NOD-SCID mice. The rates of tumor growth and final tumor size were significantly lower in mice injected with Lv-B7-H7 than in those injected with Lv-NC (**Figures 1E-G**). Thus, B7-H7 inhibition in Raji cells suppressed tumor progression *in vivo*.

### B7-H7 knockdown inhibited proliferation of the B-NHL cell line *in vitro*

**Figure 2:**
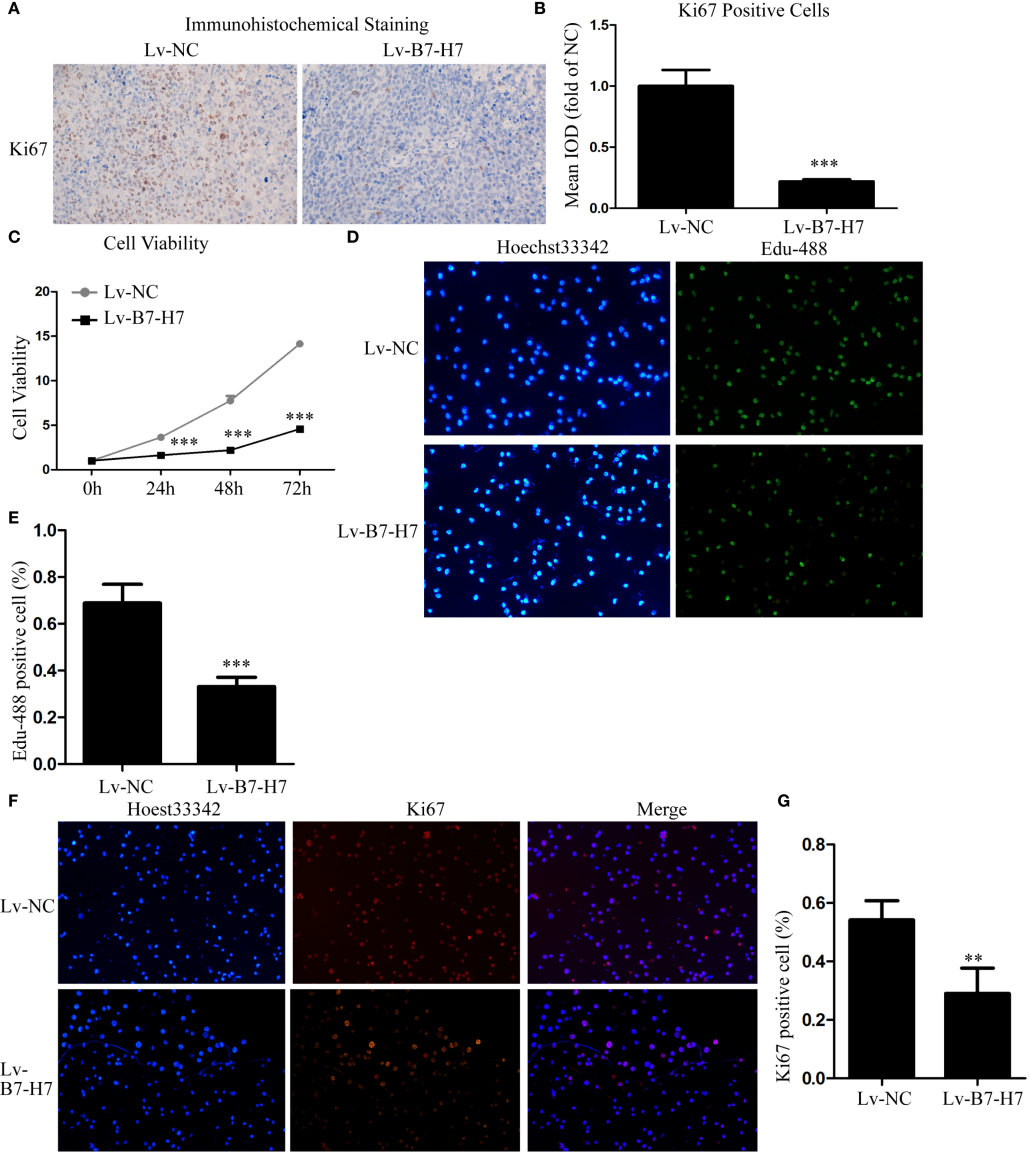
Knockdown of B7-H7 inhibited cell proliferation *in vitro.* **(A, B)** The Ki-67 expression level was detected in mouse tumor tissue sections using immunohistochemical analysis (×100), and the mean IOD was calculated. Cell proliferation ability was determined by **(C)** CCK-8 and **(D, E)** EdU-488 incorporation assays (×200) and **(F, G)** immunofluorescence analyses (×200). **p < 0.01, ***p < 0.001.

Our mouse data indicated the importance of B7-H7 expression for tumor progression in Raji cells. To further validate this conclusion, we examined the expression level of Ki-67 in mouse tumor tissues by immunohistochemical analysis. In comparison with the tumor tissue of the Lv-NC group, the Lv-B7-H7 group showed a significantly reduced degree of staining and IOD of Ki-67 (**Figures 2A, B**). Next, we performed *in vitro* validation experiments. The CCK-8 assay results showed that proliferation in the Lv-B7-H7 group was significantly inhibited (**Figure 2C**). Similar results were observed in the EdU-488 incorporation and immunofluorescence assays (**Figures 2D-G**). Thus, B7-H7 inhibition in Raji cells suppressed the proliferation of the cells *in vitro*.

### B7-H7 knockdown suppressed invasion, migration, and starvation-resistance abilities of the B-NHL cell line *in vitro*

**Figure 3:**
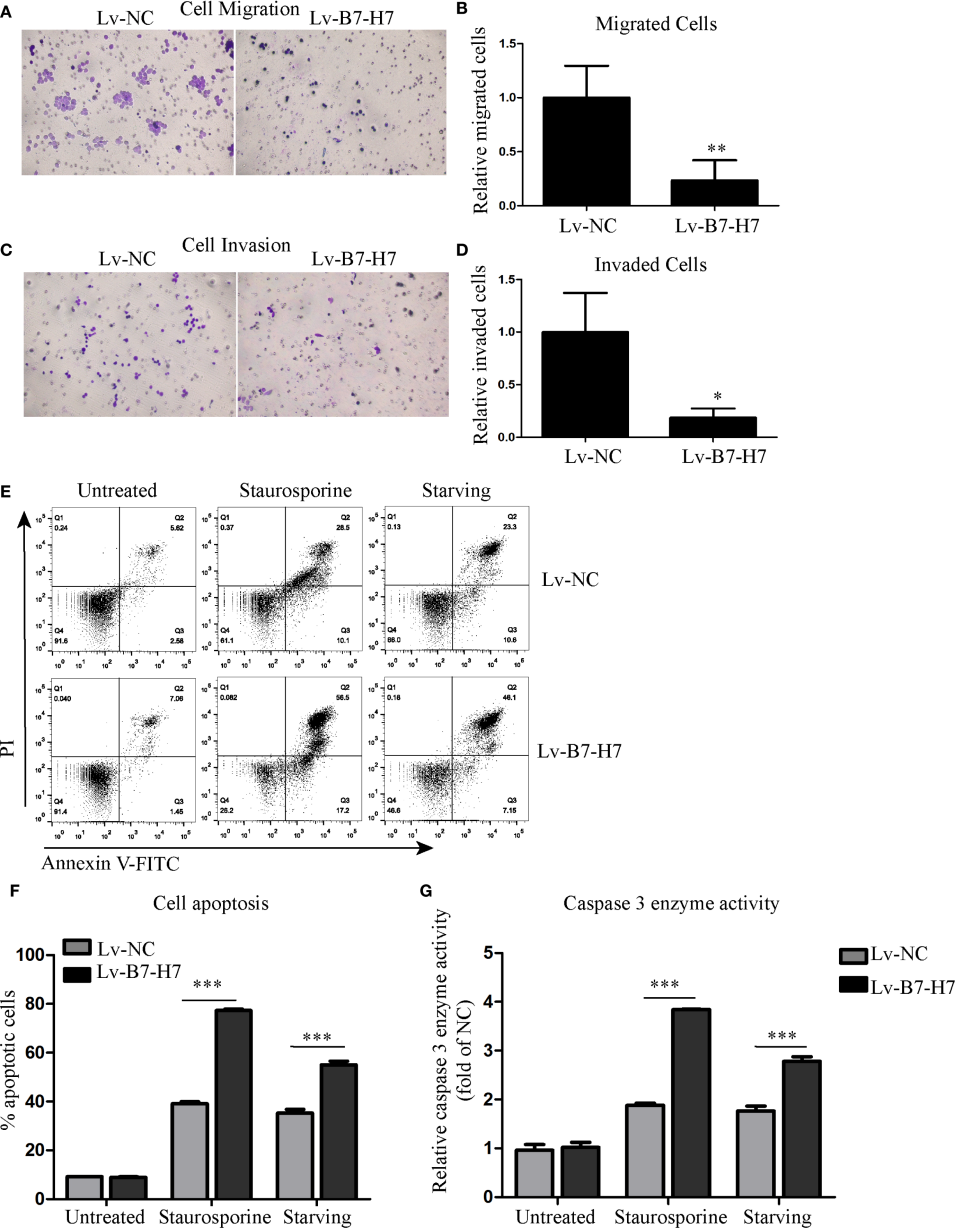
Knockdown of B7-H7 suppressed tumor characteristics. **(A-D)** Cell migration and invasion were detected using Transwell systems (×200). **(E, F)** Cell apoptosis was evaluated using the Annexin V-FITC/PI assay kit on a flow cytometer after starvation. Staurosporine (0.5 μM) was used as positive control. **(G)** The caspase 3 enzyme activity detection kit was used to evaluate cell apoptosis at the molecular level. Staurosporine (0.5 μM) was used as positive control. *p < 0.05, **p < 0.01, ***p < 0.001.

Transwell assays were performed to examine whether B7-H7 affected Raji cell migration and invasion. As shown in **Figures 3A-D**, the cell migration and invasion abilities of the Lv-B7-H7 group were lower than those of the Lv-NC group. In addition, we constructed a cell-starvation environment using a serum-free culture medium. The results showed that the starvation resistance of the Lv-B7-H7 group was inhibited in comparison with that of the Lv-NC group (**Figures 3E, F**). Furthermore, the caspase 3 enzyme activity of the Lv-B7-H7 group was elevated in comparison with that of the Lv-NC group during starvation (**Figure 3G**). Thus, B7-H7 inhibition in Raji cells decreased their invasion, migration, and starvation resistance *in vitro*.

### B7-H7 may serve as a prognostic indicator in DLBCL

**Figure 4:**
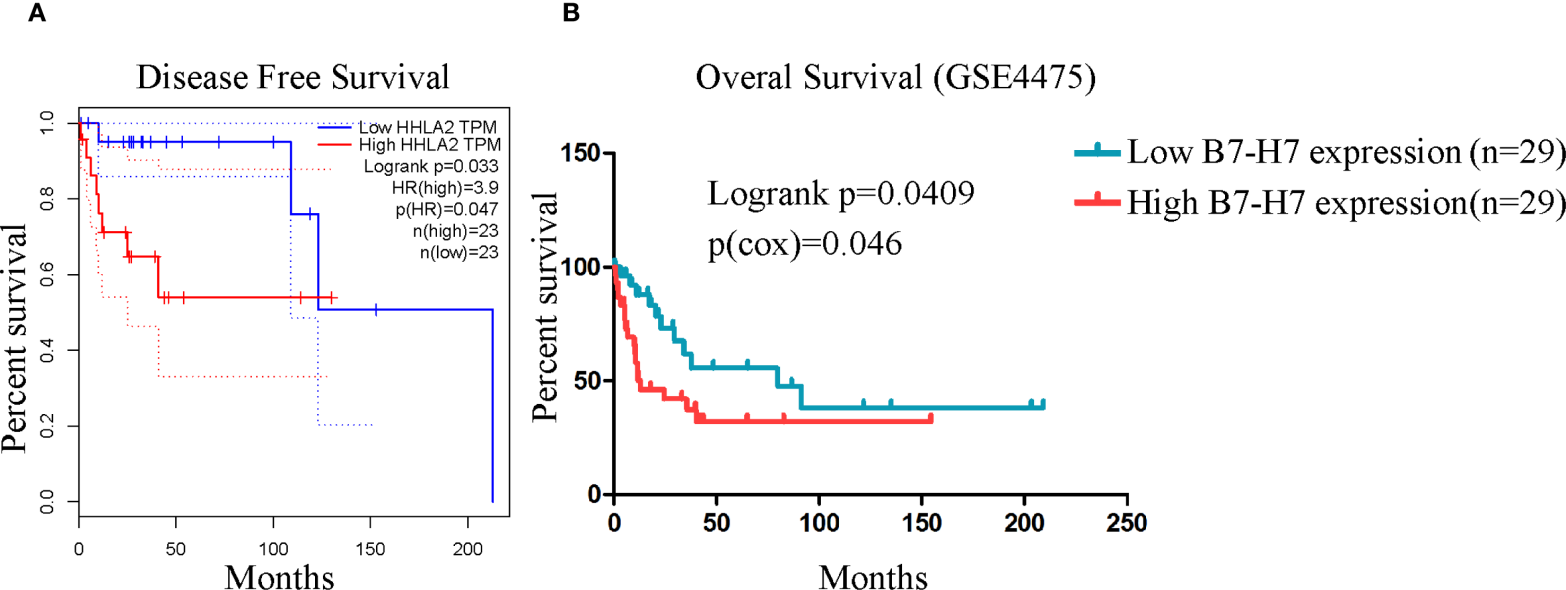
High B7-H7 expression indicated poor prognosis in analyses based on public databases. **(A)** The disease-free survival curve of DLBCL patients (n = 23/group) based on B7-H7 expression levels using the GEPIA online analysis tool. **(B)** The overall survival curve was drawn based on the survival information of the samples in the GSE4475 dataset (n = 29 in the high-expression group, and n = 29 in the low-expression group).

For further validation, we performed relevant analyses using existing databases. Since DLBCL accounts for the vast majority of B-NHL cases, we selected DLBCL samples for analysis. The GEPIA online analysis results showed that the disease-free survival time was significantly longer in the low B7-H7 expression group than in the high-expression group (**Figure 4A**). The same results were obtained using the GSE4475 dataset. High expression of B7-H7 indicated a poor prognosis (**Figure 4B**). Thus, B7-H7 may serve as a prognostic indicator for DLBCL.

### B7-H7 knockdown increased the sensitivity of Raji cells to rituximab

**Figure 5:**
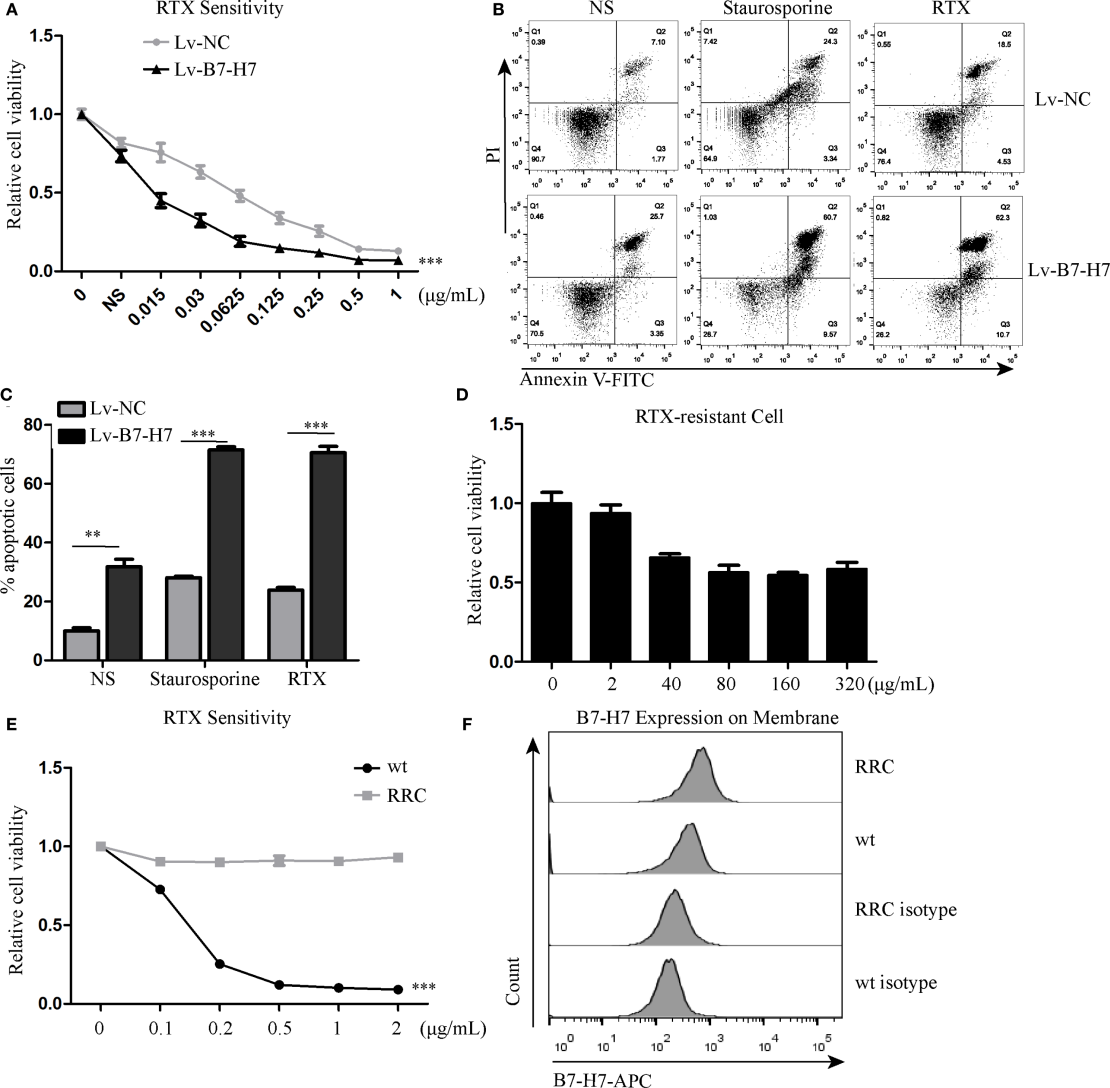
Knockdown of B7-H7 improved cell sensitivity to RTX. **(A)** Lv-NC and Lv-B7-H7 cells were incubated with increasing concentrations of RTX, and the sensitivity was detected by the CCK-8 assay at 48 h. **(B, C)** Two groups of cells were treated with 0.25 μg/mL RTX for 48 h, and cell apoptosis was detected by flow cytometry. Staurosporine (0.5 μM) was used as positive control. **(D, E)** The RTX-resistant cell line (RRC) was successfully constructed with an increasing concentration gradient. **(F)** Cell surface B7-H7 expression was detected by flow cytometry in RRC and wt cells. **p < 0.01, ***p < 0.001.

**Figure 6:**
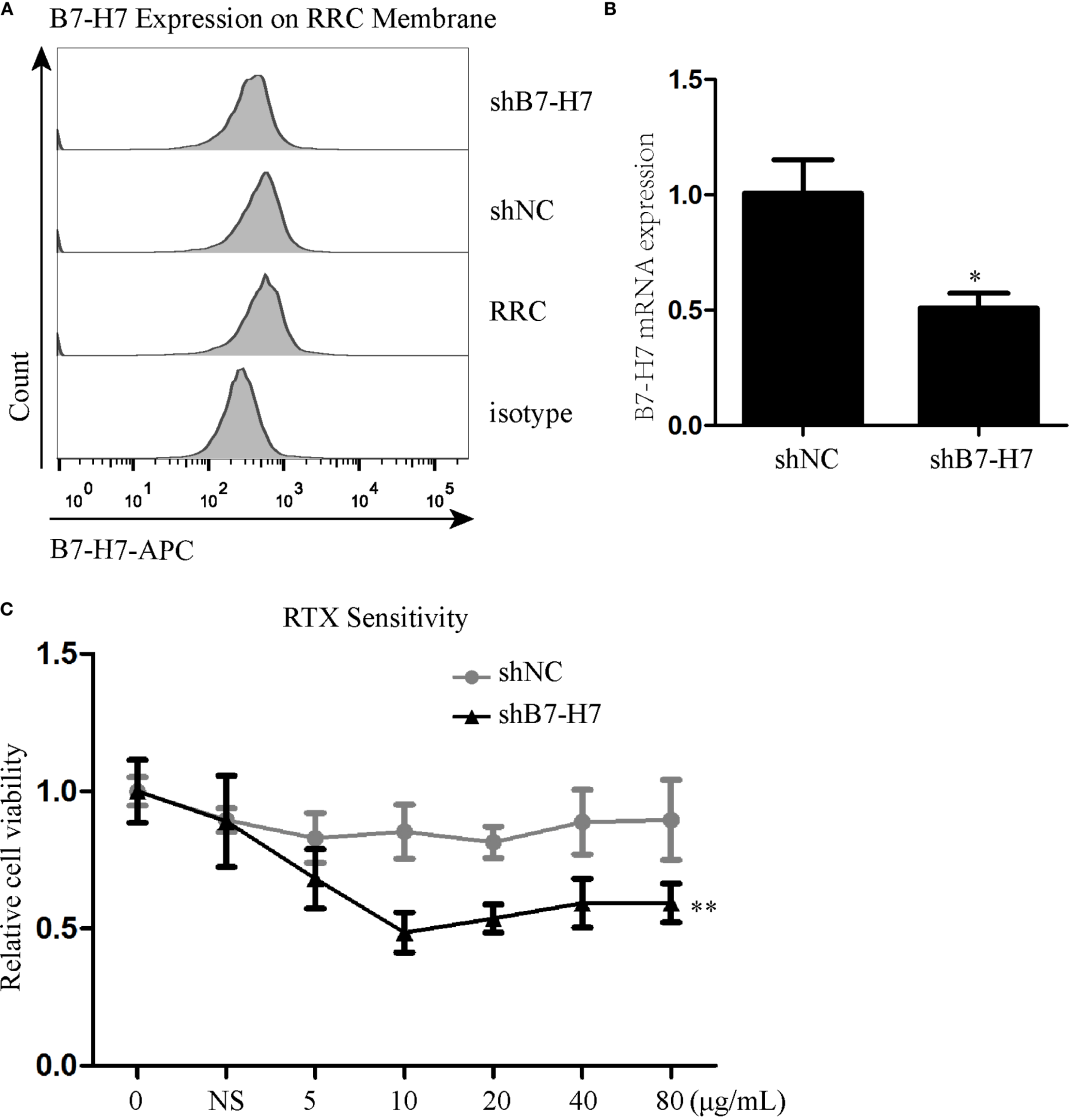
Inhibition of B7-H7 expression in the RRC weakened cell resistance to RTX. **(A, B)** B7-H7 expression level was detected by flow cytometry and qRT-PCR after transfection with B7-H7-targeting shRNA in the RRC. **(C)** RTX sensitivity was assessed by the CCK-8 assay. * *p* < 0.05, **p < 0.01.

Since B7-H7 inhibition in Raji cells decreases their proliferation, invasion, and migration abilities, we investigated the effect of B7-H7 knockdown on the cells’ sensitivity to rituximab. The lethality of rituximab at different concentrations was higher in Lv-B7-H7 cells than in the Lv-NC group (**Figures 5A-C**). To validate this finding, we successfully established an RRC, which showed strong drug resistance (**Figures 5D, E**). Surprisingly, the expression level was elevated in RRC in comparison with the wt group (**Figure 5F**). Next, we inhibited B7-H7 expression in the RRC via shRNA transcription (**Figures 6A, B**). As speculated, when the expression of B7-H7 in RRC was downregulated, the resistance of RRC was weakened (**Figure 6C**).

### RNA sequencing revealed that B7-H7 functions through the PI3K/AKT pathway

**Figure 7:**
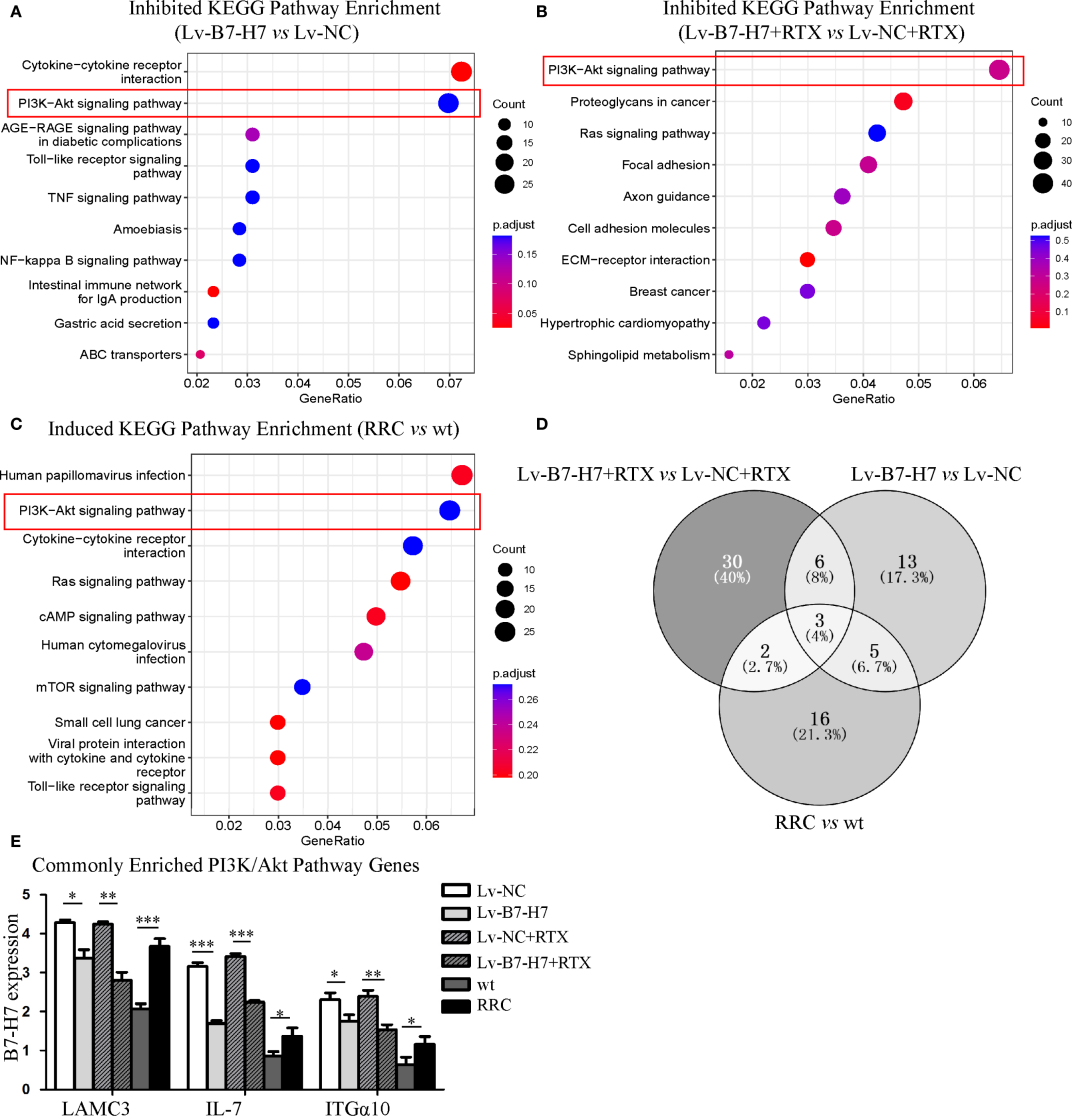
KEGG and GO enrichment analysis revealed the importance of the PI3K/Akt pathway. **(A)** The top 10 KEGG enrichment pathways of genes downregulated in the Lv-B7-H7 group in comparison with the Lv-NC group. **(B)** The top 10 KEGG enrichment pathways of genes downregulated in the Lv-B7-H7 with RTX group in comparison with the Lv-NC with RTX group. **(C)** The top 10 KEGG enrichment pathways of genes upregulated in the RRC group in comparison with the wt group. **(D)** The Venn diagram of common genes among the six groups. **(E)** The expression degrees of the three common genes in six groups of cells. *p < 0.05, **p < 0.01, ***p < 0.001.

We performed mRNA sequencing of the following six groups: Lv-B7-H7 vs. Lv-NC, Lv-B7-H7 with RTX vs. Lv-NC with RTX, and RRC vs. wt. KEGG pathway enrichment analysis showed a common pathway among the three groups, the PI3K/Akt pathway (**Figures 7A-C**). We merged and analyzed the genes enriched in the PI3K/Akt pathway in each group to identify the common genes (**Figure 7D**, **Supplementary Figure S1**). The results indicated three common genes, namely Laminin Subunit Gamma 3 (LAMC3), Interleukin 7 (IL-7), and Integrin Alpha 10 (ITGα10) (**Figure 7E**). Research has shown that these three genes are closely associated with tumor development or drug resistance. LAMC3 is linked to drug resistance in ovarian cancer, while IL-7 exerts potent immunomodulatory effects and can act on tumor cells to exert anti-tumor activity (16, 17). ITGA10 mediates the activation of the PI3K/AKT pathway, which is associated with the proliferation and chemotherapy resistance of osteosarcoma (18).

**Figure 8:**
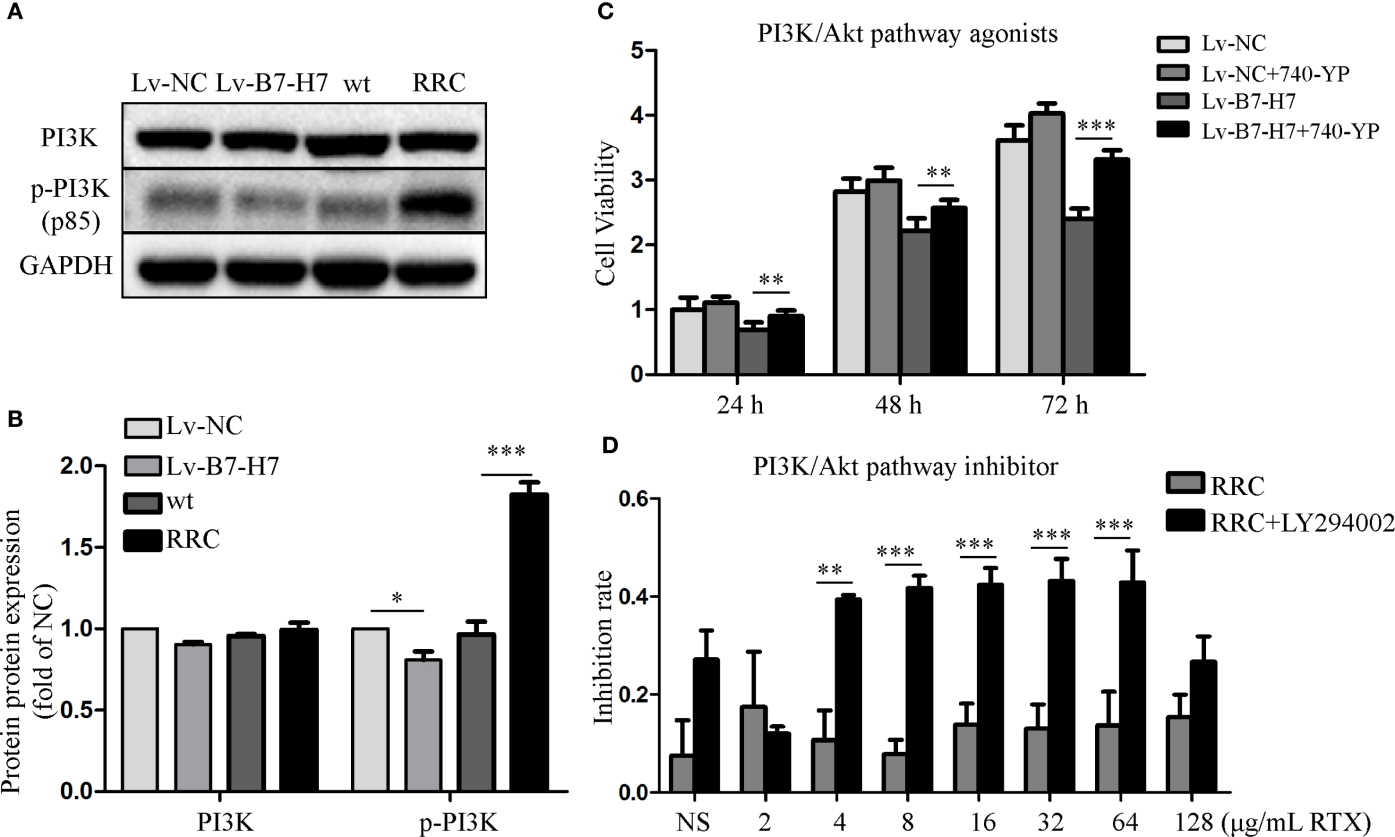
Activating or inhibiting the PI3K/Akt pathway alters the initial trend to a certain extent. **(A, B)** PI3K and its phosphorylated forms were detected by blotting. **(C)** Lv-NC and Lv-B7-H7 cells were treated with 30 μM 740-YP (agonist), and the growth rate was retested by the CCK-8 assay. **(D)** The RRC was treated with 30 μM LY294002 (inhibitor) before exposure to RTX, and the inhibition rate was determined by the CCK-8 assay. *p < 0.05, **p < 0.01, ***p < 0.001.

The blotting results showed that PI3K and its phosphorylated forms were downregulated in the Lv-B7-H7 group in comparison with the Lv-NC group (**Figures 8A, B**). These proteins were upregulated in the RRC (**Figures 8A, B**). Next, to verify the role of the PI3K/Akt pathway, we first treated Lv-NC and Lv-B7-H7 cells with the agonist 740-YP. The proliferative ability of the Lv-B7-H7 group was lower than that of the Lv-NC group; however, after treatment with 740-YP, the proliferative ability of the Lv-B7-H7 group showed a significant decrease (**Figure 8C**). Additionally, we treated the RRC with the PI3K/Akt inhibitor LY294002 and observed a decrease in drug resistance (**Figure 8D**). GO analysis revealed that when B7-H7 was knocked down, some immune-related pathways were also downregulated (**Supplementary Figure S2A, B**). After treatment with RTX in the Lv-B7-H7 and Lv-NC groups, some pathways related to tumor proliferation were downregulated in the Lv-B7-H7 + RTX group (**Supplementary Figure S2C, D**). Similar results were obtained for the drug-resistant cells (**Supplementary Figure S3**). In summary, B7-H7 knockdown may inhibit tumor progression and drug resistance by suppressing the PI3K/Akt pathway.

## Discussion

Tumor immune escape is an important characteristic of B-NHL formation, which mainly occurs through the modification of tumor cells and changes in the tumor microenvironment. Activated T lymphocytes and NK cells play crucial roles in anti-tumor immune responses (19, 20). The B7 family of co-stimulatory factor molecules provides co-stimulatory/inhibitory signals for the activation of T lymphocytes and ligands for the activation of NK cells (4). B7-H7 has attracted much attention in recent years. We reviewed the current advancements in the research on B7-H7 in tumors in a previous publication (11). The role of B7-H7 in various tumor types has been extensively discussed. The expression of B7-H7 in tumors is more common than that of programmed death ligand 1 (PD-L1), particularly in tumors with negatively expressed PD-L1 (21). Thus, immunotherapy targeting B7-H7 may have greater efficacy in patients with poor prognosis who have previously undergone PD-1/PD-L1 inhibitor treatment. However, to date, B7-H7 has only been studied in solid tumors and not in hematological tumors, especially lymphoma. Considering the complex pathogenesis and progression of blood tumors, the expression of B7-H7 in blood tumors may be lower than that in solid tumors.

The present study is the first to investigate the prospective role of B7-H7 in B-NHL. For this purpose, we established a B7-H7 knockdown cell line at a preliminary stage. A significant decrease in tumor growth, migration, invasion, and starvation tolerance abilities was observed in the Lv-B7-H7 group in the mouse model and *in vitro* functional experiments. These abilities of tumors are important factors in maintaining their malignant growth, and high expression of B7-H7 in B-NHLs enhanced these abilities in tumors. The high expression of surface B7-H7 in tumor cells can continuously activate signaling pathways that maintain tumor biological behavior within the cells, thereby promoting tumor progression. To explore whether B7-H7 on the surface of B-NHL tumor cells activates specific intracellular pathways, RNA-seq analysis was performed. The PI3K/Akt pathway was found to be downregulated in the Lv-B7-H7 group. After preliminary experimental verification, we speculated that elevated expression of B7-H7 may promote B-NHL tumor progression by activating the PI3K/Akt pathway. Further mechanistic research was lacking, especially on the key elements of B7-H7 that activate the PI3K/AKT pathway and trigger cascade reactions. Studies on the mechanisms of other tumors have found that B7-H7 may exert pro-tumor effects by activating the Janus kinase (JAK)/signal transducer and activator of transcription (STAT), PI3K/Akt, and epidermal growth factor receptor (EGFR)/mitogen-activated protein kinase (MAPK)/ERK pathways (12, 22, 23). However, further research has not been conducted. Evidence has shown that B7-H7 upregulates genes related to the PI3K/Akt signaling pathway (12).

To investigate the clinical significance of B7-H7, the sample sequencing data of patients with B-NHL in public databases were analyzed. These results indicated that patients with high B7-H7 expression had lower survival rates. The degree of B7-H7 expression in the samples showing relapse was higher than that in the relapse-free samples. These data indicate that B7-H7 may serve as a potential guide for the prognosis of clinical patients. Surface B7-H7 of B-NHL cells may bind to receptors on T cells to evade immune surveillance, leading to the failure of anticancer therapy. Potential approaches to disrupt this process, such as blocking the binding of B7-H7 to its receptor, should be investigated in future experiments. B7-H7 has been proven to serve as a standalone predictor of prognosis in patients with multiple solid tumors, including lung cancer, gastric cancer, pancreatic cancer, liver cancer, and oral cancer (6–11). B7-H7 has also been shown to play crucial roles in cancer development, suppressing immune surveillance, disrupting T-cell anti-tumor immunity, and promoting immune evasion (24, 25). B7-H7 expressed on the surface of tumor cells may promote tumor immune escape by interacting with receptors on activated T cells, thereby promoting the occurrence and development of tumors (12). Inflammatory factors activate B7-H7 in dendritic cells and macrophages, trigger tumor antigen-specific T-cell apoptosis, inhibit T-cell immune responses in the tumor microenvironment, and promote tumor immune escape (26). The activation of B7-H7 expression induces M2 polarization and chemotactic migration of macrophages, leading to immune escape and tumor development (23, 27).

In comparison with the use of CHOP alone, R-CHOP has been shown to increase the overall survival rate of patients with B-NHL by 10%-15% (28). However, approximately 50% of patients initially do not respond to this regimen, and the majority eventually develop resistance to further treatment with RTX (2). Moreover, treatment for patients with relapsing/refractory B-NHL after receiving the R-CHOP regimen is extremely difficult. However, the mechanisms underlying the development of resistance remain unclear. Thus, understanding the mechanisms of resistance is crucial for improving clinical outcomes. In this study, we found that the Lv-B7-H7 group was more sensitive to RTX, which is an encouraging finding. The possible reasons for this are worth investigating in-depth. Since rituximab is a monoclonal antibody targeting CD20, any abnormalities in CD20 protein expression, such as downregulation, alterations in binding site structure, or changes in the cell membrane (such as lipid raft domain recombination), can lead to rituximab resistance (28–30). In our study, we did not observe any alterations in CD20 expression after B7-H7 knockdown (data not shown). Therefore, B7-H7 may affect RTX resistance through other mechanisms. To further explore these mechanisms, we constructed an RTX-resistant strain. Increased B7-H7 expression was observed in the resistant strain. Resistance to RTX was slightly reversed following inhibition of B7-H7 expression in drug-resistant strains. Thus, B7-H7 on the surface of B-NHL cells can be considered to promote tumor progression and induce drug resistance. RNA sequencing was also performed on the resistant strains. The PI3K/Akt pathway was upregulated in the resistant strains. On the basis of these results, we reasonably speculate that recurrent/refractory B-NHL cells show high expression of B7-H7, which activates the intracellular PI3K/AKT pathway, leading to the promotion of tumor progression and induction of resistance to RTX. Extensive clinical studies have been conducted on the combination of RTX and other anticancer agents (31). Activation of the PI3K/Akt signaling pathway is achieved through the sequential phosphorylation of PI3K, PIP2, and Akt. Akt directly activates nuclear factor kappa B (NF-κB) signaling through phosphorylated IκBs, thereby affecting cell survival, proliferation, invasion, angiogenesis, and chemotherapy resistance (32). The PI3K/Akt pathway is a key factor in the emergence of R-CHOP resistance, and high levels of phosphorylated Akt docking adversely affect the prognosis of patients treated with R-CHOP (33). In patients with PI3K/Akt/mTOR activation, the clinical course deteriorates faster, treatment response is poor, and survival time is shortened (33). The clinical course of PI3K/Akt/mTOR activated patients deteriorates faster, treatment response is poor, and survival time is shortened (33). The addition of PI3K inhibitors to the R-CHOP treatment regimen has been shown to completely inhibit tumor growth in R-CHOP-resistant cells (34). Additionally, genes related to the PI3K/AKT pathway also play a critical role in tumor immune evasion and drug resistance. Our sequencing results have identified three PI3K/AKT pathway-related genes: LAMC3, IL-7, and ITGA10. Studies have shown that interfering with LAMC3 expression can reduce the carboplatin resistance of ovarian cancer cells (16). IL-7 is essential for the development, maintenance, and proliferation of T lymphocytes, highlighting its potential role as an adjuvant in cancer vaccine development (35). IL-7 also exerts tumor-promoting effects by activating the downstream PI3K-AKT pathway and has been utilized in clinical research (36). ITGA10 stimulates the PI3K/AKT signaling pathway and drives the proliferation and chemotherapy resistance of osteosarcoma cells (18). These three genes are precisely our starting point for further research into the PI3K/AKT signaling pathway. Thus, the combination of RTX and inhibition of the PI3K/Akt/mTOR pathway may be a promising treatment strategy for B-NHL (33).

B7-H7 has been extensively studied in other solid tumors; however, research on B7-H7 in hematological tumors is lacking. Moreover, the expression and activation mechanisms of B7-H7 in the emergence, progression, and drug resistance of B-NHL remain unclear. Our study focused on the clinical challenge of poor immunotherapy efficacy and frequent drug resistance in patients with B-NHL and aimed to explore the mechanisms underlying rapid tumor progression in B-NHL and the key factors for drug resistance in B-NHL. Furthermore, our study has several limitations. These include the lack of investigations into the interaction between B7-H7 and its receptors, as well as co-culture studies of immune cells with tumor cells. Additionally, the combined therapeutic effects of PI3K/AKT pathway modulation with rituximab have not been validated in murine models. Moreover, the tumor types analyzed were relatively homogeneous, with insufficient exploration of molecular subtypes. The aforementioned limitations warrant further validation in our subsequent studies. In summary, we speculate that the PI3K/Akt signaling pathway is a crucial pathway that induces B-NHL tumor proliferation and drug resistance after B7-H7 activation. Targeted disruption of the B7-H7-PI3K/Akt regulatory axis may inhibit cell proliferation and tumor metastasis, increase sensitivity to RTX, and thereby facilitate the treatment of patients with B-NHL. The combination of B7-H7-PI3K/Akt interference with RTX may significantly improve drug efficacy and provide substantial economic and social benefits.

## Data Availability

The raw data supporting the conclusions of this article will be made available by the authors, without undue reservation.

## References

[1] Ma MCJ Tadros S Bouska A Heavican T Yang H Deng Q. Subtype-specific and co-occurring genetic alterations in B-cell non-Hodgkin lymphoma. Haematologica. (2022) 107:690–701. doi: 10.3324/haematol.2020.274258, PMID: 33792219 PMC8883549

[2] Zhou N Choi J Grothusen G. DLBCL-associated NOTCH2 mutations escape ubiquitin-dependent degradation and promote chemoresistance. Blood. (2023) 142:973–88. doi: 10.1182/blood.2022018752, PMID: 37235754 PMC10656726

[3] Szeto C Zareie P. Covalent TCR-peptide-MHC interactions induce T cell activation and redirect T cell fate in the thymus. Nat Commun. (2022) 13:4951. doi: 10.1038/s41467-022-32692-4, PMID: 35999236 PMC9399087

[4] Zhang W Qiu Y Xie X Fu Y Wang L Cai Z. B7 family members in lymphoma: promising novel targets for tumor immunotherapy? Front Oncol. (2021) 11:647526. doi: 10.3389/fonc.2021.647526, PMID: 33869045 PMC8044412

[5] Janakiram M Shah UA Liu W Zhao A Schoenberg MP Zang X. The third group of the B7-CD28 immune checkpoint family: HHLA2, TMIGD2, B7x, and B7-H3. Immunol Rev. (2017) 276:26–39. doi: 10.1111/imr.12521, PMID: 28258693 PMC5338461

[6] Wei L Tang L Chang H Huo S Li Y. HHLA2 overexpression is a novel biomarker of Malignant status and poor prognosis in gastric cancer. Hum Cell. (2020) 33:116–22. doi: 10.1007/s13577-019-00280-2, PMID: 31552567

[7] Farrag MS Ibrahim EM El-Hadidy TA Akl MF Elsergany AR Abdelwahab HW. Human endogenous retrovirus-H long terminal repeat- associating protein 2 (HHLA2) is a novel immune checkpoint protein in lung cancer which predicts survival. Asian Pac J Cancer Prev. (2021) 22:1883–9. doi: 10.31557/APJCP.2021.22.6.1883, PMID: 34181347 PMC8418860

[8] Zhu Y Chen J Liu Y Zheng X Feng J Chen X. Prognostic values of B7-H3, B7-H4, and HHLA2 expression in human pancreatic cancer tissues based on mIHC and spatial distribution analysis. Pathol Res Pract. (2022) 234:153911. doi: 10.1016/j.prp.2022.153911, PMID: 35489125

[9] Ding L Yu Q Yang S Yang WJ Liu T Xian JR. Comprehensive analysis of HHLA2 as a prognostic biomarker and its association with immune infiltrates in hepatocellular carcinoma. Front Immunol. (2022) 13:831101. doi: 10.3389/fimmu.2022.831101, PMID: 35371079 PMC8968642

[10] Xiao Y Li H Yang LL Mao L Wu CC Zhang WF. The expression patterns and associated clinical parameters of human endogenous retrovirus-H long terminal repeat-associating protein 2 and transmembrane and immunoglobulin domain containing 2 in oral squamous cell carcinoma. Dis Markers. (2019) 2019:5421985. doi: 10.1155/2019/5421985, PMID: 31089395 PMC6476002

[11] Su Q Du J Xiong X Xie X Wang L. B7-H7: A potential target for cancer immunotherapy. Int Immunopharmacol. (2023) 121:110403. doi: 10.1016/j.intimp.2023.110403, PMID: 37290327

[12] Rieder SA Wang J White N Qadri A Menard C Stephens G. B7-H7 (HHLA2) inhibits T-cell activation and proliferation in the presence of TCR and CD28 signaling. Cell Mol Immunol. (2021) 18:1503–11. doi: 10.1038/s41423-020-0361-7, PMID: 32005952 PMC8166953

[13] Rougé L Chiang N Steffek M. Structure of CD20 in complex with the therapeutic monoclonal antibody rituximab. Science. (2020) 367:1224–30. doi: 10.1126/science.aaz9356, PMID: 32079680

[14] Kupcova K Senavova J Jura F Herman V Rajmonova A Pacheco-Blanco M. Vertical targeting of the PI3K/AKT pathway at multiple points is synergistic and effective for non-Hodgkin lymphoma. Exp Hematol Oncol. (2024) 13:108. doi: 10.1186/s40164-024-00568-6, PMID: 39487517 PMC11529427

[15] Che F Xie X Wang L Su Q Jia F Ye Y. B7-H6 expression is induced by lipopolysaccharide and facilitates cancer invasion and metastasis in human gliomas. Int Immunopharmacol. (2018) 59:318–27. doi: 10.1016/j.intimp.2018.03.020, PMID: 29679856

[16] Liu X Zhang J Xia L Su Y Chen X Wang C. LAMC3 interference reduces drug resistance of carboplatin-resistant ovarian cancer cells. Sci Rep. (2025) 15:20399. doi: 10.1038/s41598-025-07055-w, PMID: 40593083 PMC12217247

[17] Wang C Kong L Kim S Lee S Oh S. The role of IL-7 and IL-7R in cancer pathophysiology and immunotherapy. Int J Mol Sci. (2022) 23:10412. doi: 10.3390/ijms231810412, PMID: 36142322 PMC9499417

[18] Li H Shen X Ma M Liu W Yang W Wang P. ZIP10 drives osteosarcoma proliferation and chemoresistance through ITGA10-mediated activation of the PI3K/AKT pathway. Int J Mol Sci. (2021) 40:340. doi: 10.1186/s13046-021-02146-8, PMID: 34706747 PMC8549349

[19] Tang F Li J Qi L Liu D Bo Y Qin S. A pan-cancer single-cell panorama of human natural killer cells. Cell. (2023) 186:4235–4251.e4220. doi: 10.1016/j.cell.2023.07.034, PMID: 37607536

[20] Liu X Zhang W Han Y Cheng H Liu Q Ke S. FOXP3(+) regulatory T cell perturbation mediated by the IFNγ-STAT1-IFITM3 feedback loop is essential for anti-tumor immunity. Nat Commun. (2024) 15:122. doi: 10.1038/s41467-023-44391-9, PMID: 38167862 PMC10761945

[21] Wang B Ran Z Liu M Ou Y. Prognostic significance of potential immune checkpoint member HHLA2 in human tumors: A comprehensive analysis. Front Immunol. (2019) 10:1573. doi: 10.3389/fimmu.2019.01573, PMID: 31379814 PMC6644528

[22] Wei Y Ren X Galbo PM Jr. KIR3DL3-HHLA2 is a human immunosuppressive pathway and a therapeutic target. Sci Immunol. (2021) 6 (61):eabf9792. doi: 10.1126/sciimmunol.abf9792, PMID: 34244312 PMC9744578

[23] Sun W Li S Tang G Sun S Luo Y Bai R. HHLA2 deficiency inhibits non-small cell lung cancer progression and THP-1 macrophage M2 polarization. Cancer Med. (2021) 10:5256–69. doi: 10.1002/cam4.4081, PMID: 34152094 PMC8335813

[24] Janakiram M Chinai JM Zhao A Sparano JA Zang X. HHLA2 and TMIGD2: new immunotherapeutic targets of the B7 and CD28 families. Oncoimmunology. (2015) 4:e1026534. doi: 10.1080/2162402X.2015.1026534, PMID: 26405587 PMC4570140

[25] Chen L Zhu D Feng J Zhou Y Wang Q Feng H. Overexpression of HHLA2 in human clear cell renal cell carcinoma is significantly associated with poor survival of the patients. Cancer Cell Int. (2019) 19:101. doi: 10.1186/s12935-019-0813-2, PMID: 31015801 PMC6469208

[26] Li Y Lv C Yu Y Wu B Zhang Y Lang Q. KIR3DL3-HHLA2 and TMIGD2-HHLA2 pathways: The dual role of HHLA2 in immune responses and its potential therapeutic approach for cancer immunotherapy. J Adv Res. (2023) 47:137–50. doi: 10.1016/j.jare.2022.07.013, PMID: 35933091 PMC10173190

[27] Wang R Guo H Tang X Zhang T Liu Y Zhang C. Interferon gamma-induced interferon regulatory factor 1 activates transcription of HHLA2 and induces immune escape of hepatocellular carcinoma cells. Inflammation. (2022) 45:308–30. doi: 10.1007/s10753-021-01547-3, PMID: 34536158

[28] Guan XW Wang HQ Ban WW Chang Z Chen HZ Jia L. Novel HDAC inhibitor Chidamide synergizes with Rituximab to inhibit diffuse large B-cell lymphoma tumour growth by upregulating CD20. Cell Death Dis. (2020) 11:20. doi: 10.1038/s41419-019-2210-0, PMID: 31907371 PMC6944697

[29] Shimizu R Kikuchi J Wada T Ozawa K Kano Y Furukawa Y. HDAC inhibitors augment cytotoxic activity of rituximab by upregulating CD20 expression on lymphoma cells. Leukemia. (2010) 24:1760–8. doi: 10.1038/leu.2010.157, PMID: 20686505

[30] Scialdone A Khazaei S Hasni MS Lennartsson A Gullberg U Drott K. Depletion of the transcriptional coactivators CREB-binding protein or EP300 downregulates CD20 in diffuse large B-cell lymphoma cells and impairs the cytotoxic effects of anti-CD20 antibodies. Exp Hematol. (2019) 79:35–46.e31. doi: 10.1016/j.exphem.2019.10.004, PMID: 31669559

[31] Lv Y Du Y Li K Ma X Wang J Du T. The FACT-targeted drug CBL0137 enhances the effects of rituximab to inhibit B-cell non-Hodgkin’s lymphoma tumor growth by promoting apoptosis and autophagy. Cell Commun Signal. (2023) 21:16. doi: 10.1186/s12964-022-01031-x, PMID: 36691066 PMC9869543

[32] Xu HB Chen XZ Yu ZL Xue F. Guggulsterone from Commiphora mukul potentiates anti-glioblastoma efficacy of temozolomide *in vitro* and *in vivo* via down-regulating EGFR/PI3K/Akt signaling and NF-κB activation. J Ethnopharmacol. (2023) 301:115855. doi: 10.1016/j.jep.2022.115855, PMID: 36280019

[33] Wang L Li LR. R-CHOP resistance in diffuse large B-cell lymphoma: biological and molecular mechanisms. Chin Med J (Engl). (2020) 134:253–60. doi: 10.1097/CM9.0000000000001294, PMID: 33323828 PMC7846449

[34] Chen J Ge X Zhang W Ding P Du Y Wang Q. PI3K/AKT inhibition reverses R-CHOP resistance by destabilizing SOX2 in diffuse large B cell lymphoma. Theranostics. (2020) 10:3151–63. doi: 10.7150/thno.41362, PMID: 32194860 PMC7053184

[35] Zhao Y Wei K Chi H Xia Z Li X. IL-7: A promising adjuvant ensuring effective T cell responses and memory in combination with cancer vaccines? Front Immunol. (2022) 13:1022808. doi: 10.3389/fimmu.2022.1022808, PMID: 36389666 PMC9650235

[36] Nakao S Arai Y Tasaki M Yamashita M. Intratumoral expression of IL-7 and IL-12 using an oncolytic virus increases systemic sensitivity to immune checkpoint blockade. Sci Transl Med. (2020) 12 (526):eaax7992. doi: 10.1126/scitranslmed.aax7992W, PMID: 31941828

