# Supplementary material for: B7-H7 knockdown suppresses the proliferation, metastasis, and drug resistance of B-cell non-Hodgkin lymphoma cells by inhibiting the PI3K/Akt pathway
Source: Front Oncol. 2025 Oct 16;15:1665309. doi: 10.3389/fonc.2025.1665309 (PMC12571572; doi:10.3389/fonc.2025.1665309)
Supplement: Supplementary file 1 [file DataSheet1.pdf]

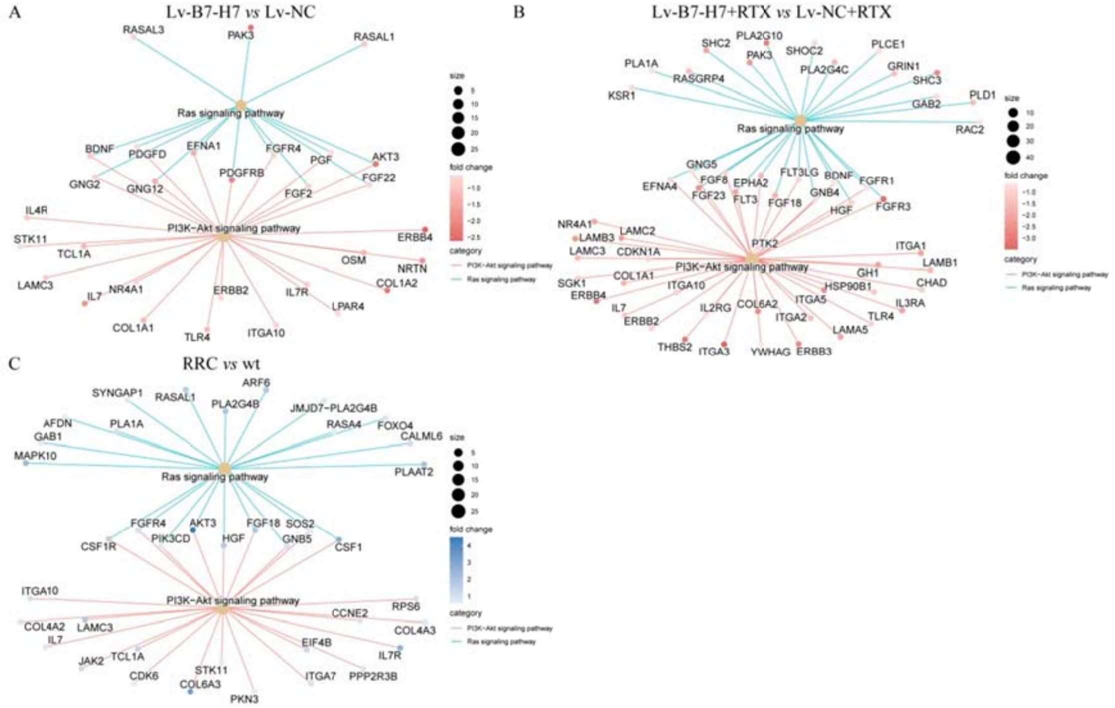

Figure S1. The network diagram of PI3K/Akt and Ras signaling pathway

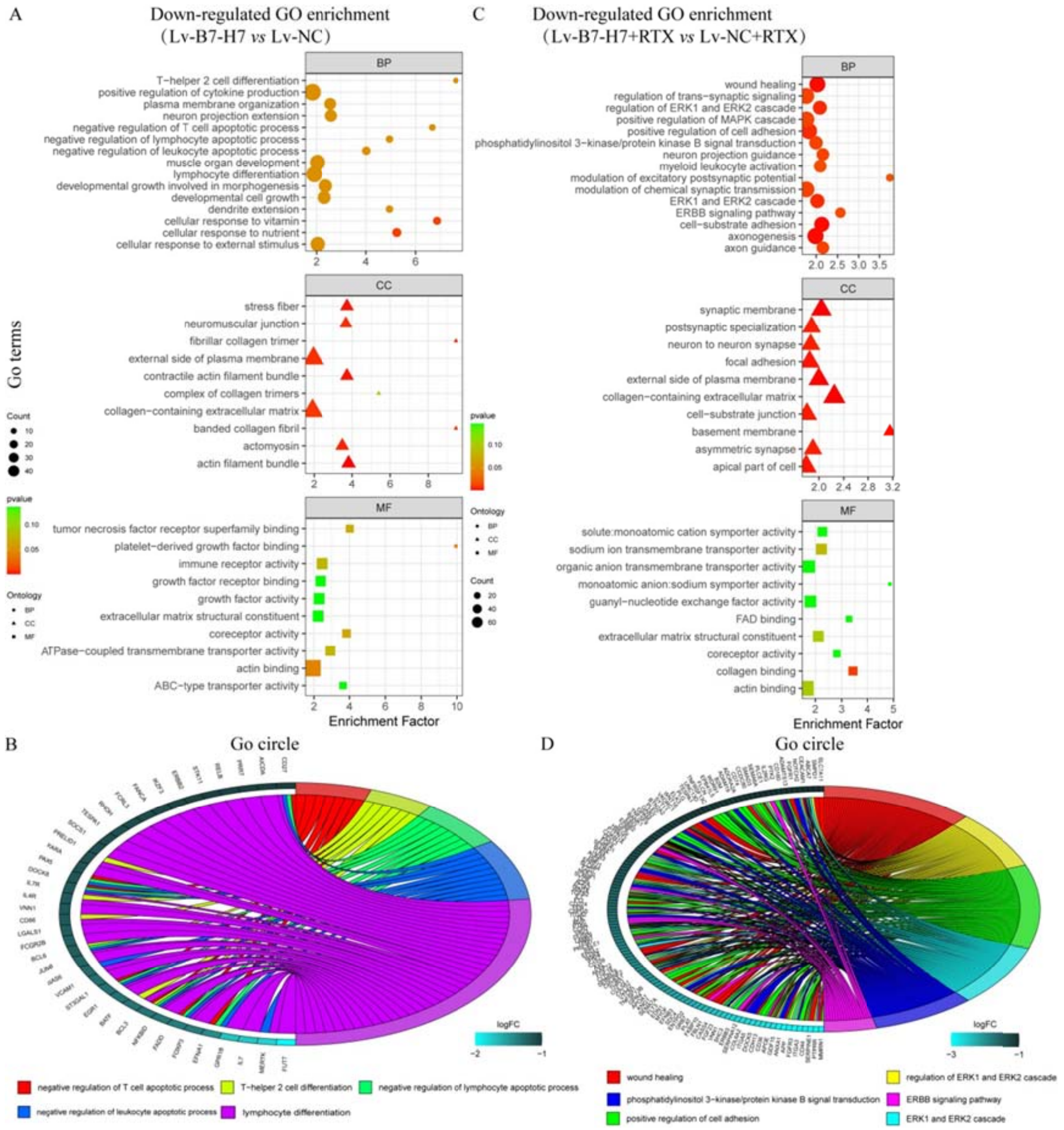

Figure S2. Go enrichment analysis of four groups

(A) Go enrichment of downregulated genes of Lv-B7-H7 group compared with Lv-NC group. (B) Five immune-related pathways were selected for GO circle mapping. (C) Go enrichment of downregulated genes of Lv-B7-H7 with RTX group compared with Lv-NC with RTX group. (D) Six pathways related to tumor progression were selected for GO circle mapping.

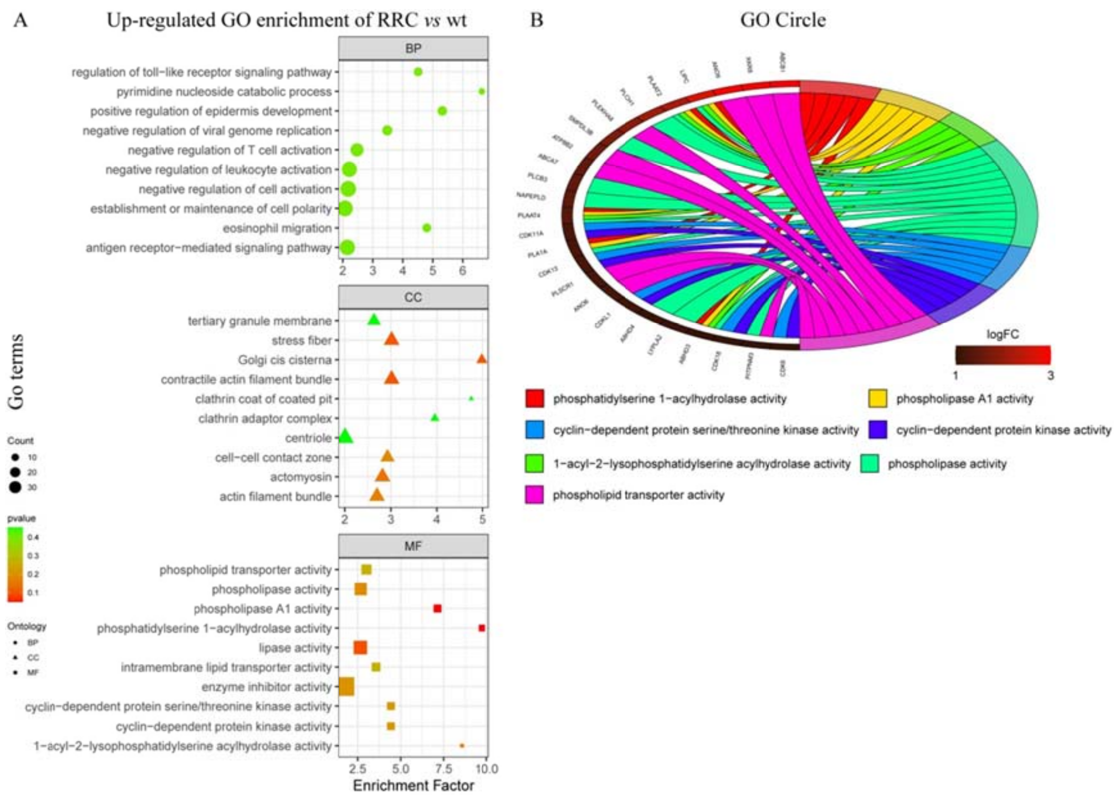

Figure S3. Go enrichment analysis of RRC *vs* wt

(A) Go enrichment of upregulated genes of RRC compared with wt group. (B) Related to tumor progression were selected for GO circle mapping.
